# Intracellular Fate of Universally Labelled ^13^C Isotopic Tracers of Glucose and Xylose in Central Metabolic Pathways of *Xanthomonas oryzae*

**DOI:** 10.3390/metabo8040066

**Published:** 2018-10-15

**Authors:** Manu Shree, Shyam K. Masakapalli

**Affiliations:** School of Basic Sciences, Indian Institute of Technology Mandi, Kamand 175005, HP, India; manushree.iitmandi@gmail.com

**Keywords:** *Xanthomonas oryzae*, mass isotopomer distribution, GC-MS, pathway mapping, PPP, ED, TCA, ^13^C-MFA, metabolic flux analysis, TBDMS derivatization, natural isotope correction

## Abstract

The goal of this study is to map the metabolic pathways of poorly understood bacterial phytopathogen, *Xanthomonas oryzae* (Xoo) BXO43 fed with plant mimicking media XOM2 containing glutamate, methionine and either 40% [^13^C_5_] xylose or 40% [^13^C_6_] glucose. The metabolic networks mapped using the KEGG mapper and the mass isotopomer fragments of proteinogenic amino acids derived from GC-MS provided insights into the activities of Xoo central metabolic pathways. The average ^13^C in histidine, aspartate and other amino acids confirmed the activities of PPP, the TCA cycle and amino acid biosynthetic routes, respectively. The similar labelling patterns of amino acids (His, Ala, Ser, Val and Gly) from glucose and xylose feeding experiments suggests that PPP would be the main metabolic route in Xoo. Owing to the lack of annotated gene phosphoglucoisomerase in BXO43, the ^13^C incorporation in alanine could not be attributed to the competing pathways and hence warrants additional positional labelling experiments. The negligible presence of ^13^C incorporation in methionine brings into question its potential role in metabolism and pathogenicity. The extent of the average ^13^C labelling in several amino acids highlighted the contribution of pre-existing pools that need to be accounted for in ^13^C-flux analysis studies. This study provided the first qualitative insights into central carbon metabolic pathway activities in Xoo.

## 1. Introduction

*Xanthomonas oryzae* pv. *oryzae* (Xoo), the causal agent of rice bacterial blight, is among the top ten bacterial phytopathogens that contribute to crop loss [1,2,3]. The genus is also well-known to be an effective host for the industrial production of Xanthan, a natural thickening agent used in salad dressings, sauces, gravies, dairy products and desserts, etc. [4,5]. The significance of Xoo led researchers to undertake studies to understand its biology at the level of genome [1,3], Proteome [6,7] and Transcriptome [8]. Research interests also lie in understanding the involvement of carbon metabolic pathways in the virulence of agricultural phytopathogens [9,10]. In general, the expression of hypersensitive and pathogenicity response genes (hrp) is a direct indication of bacterial virulence [11]. In *Xanthomonas oryzae* pv. *oryzae*, XOM2 media is known to be an efficient hrp inducer [12] and is termed as plant mimic media mainly containing glutamate, xylose and methionine as potential carbon or nitrogen sources. No studies involving ^13^C tracers have been reported on Xoo, which comprehensively map the central metabolic pathways and would assist in defining metabolic phenotypes [13] under different nutritional regimes. This study aims to evaluate the ^13^C tracer analysis to map the central carbon metabolism of Xoo.

Stable isotope (^13^C) labelled substrate (glucose and xylose) feeding of microbial systems such as Xoo will define metabolic phenotypes when subjected to a steady state metabolic flux analysis (MFA), a powerful approach to quantify multiple intracellular fluxes through the central metabolic pathways [14]. The flux maps quantify the carbon and energy metabolism that could evaluate the metabolic performance of bacterial strains, which provides detailed cellular activities. Care must be taken to first evaluate and fulfil the experimental and modelling pre-requisites. This relies on the assumption that the system is in a metabolic and isotopic steady state, at which point the isotopic label redistribution within metabolites is measured, followed by subsequent modelling to determine the in vivo fluxes across the metabolic network [15]. As Xoo is a slow-growing microbe, it is necessary to evaluate the feasibility of ^13^C tracer-based studies to generate comprehensive flux maps.

In this study, we explored the ^13^C label incorporation in proteinogenic amino acids of Xoo (BXO43) to map its central metabolic pathway activities qualitatively under plant mimic XOM2 media [7,8]. The mass isotopomers of amino acid fragments were validated for their potential use for further analysis. Tsuge et al., 2004, have reported that a *Xanthomonas oryzae* mutant that could not grow in glucose has grown in rice leaves, indicating that glucose is not essential for its pathogenicity whereas the presence of xylose is found to be an essential factor for hrp gene induction and thus ensures pathogenicity. We thus subjected the Xoo cells to either 40% [^13^C_6_] glucose or 40% [^13^C_5_] xylose to investigate the average label incorporation in protein-derived amino acids in the presence of different carbon tracers [16] (see Supplementary Figure S1 for the experimental workflow). The study provided insights into the central carbon metabolic pathways of Xoo. The identified valid mass isotopomer fragments of amino acids along with the workflow can assist in undertaking a steady-state ^13^C-MFA. The tracer-based metabolic pathway study of slow-growing bacterial phytopathogen would be indispensable for future studies directed at crop management and food security. 

## 2. Results

### 2.1. Central Metabolic Pathway Mapping of BXO43 Strain

**Figure 1:**
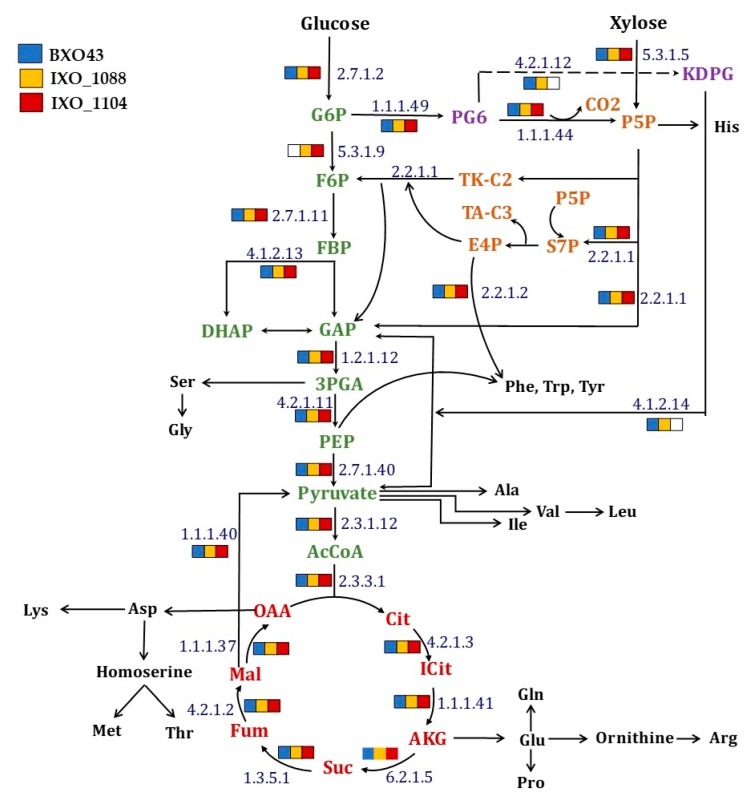
An overview of the simple central carbon network of the *Xanthomonas oryzae* pv. *oryzae* BXO43 strain. A KEGG mapper was used for mapping the presence and absence of any annotated enzyme throughout the metabolic pathways among the Xoo strains (see legend for color codes). The enzyme responsible at each node is represented by enzyme commission (EC) numbers. The absence of any enzyme in a strain is shown by the absence of any color. The central metabolic pathway intermediates that are responsible for various amino acids biosynthetic routes are presented. The intermediates of four major metabolic pathways, namely, Glycolysis, the Tricarboxylic acid cycle, Entner-Doudoroff and pentose phosphate pathway (simplified) are shown in green, red, violet and orange, respectively, whereas amino acids are depicted using their three-letter code in black.

The analysis of central metabolic pathways in wild-type (BXO43) and highly virulent (IXO_1088, IXO_1104) *Xanthomonas oryzae* strains reconstructed by the KEGG pathway mapper confirmed the TCA (Tricarboxylic acid) cycle, PPP (pentose phosphate pathway) and all the amino acid biosynthetic pathways (Figure 1). In the case of glycolysis, the gene coding for phosphoglucoisomerase (E.C 5.3.1.9) was not annotated in the Xoo BXO43 strain, while it was intact in the other two pathogenic strains studied (Figure 1). The inherent absence of the key glycolytic gene raises interesting queries in relation to mapping the flow of carbon through the central metabolic pathways. In fact, earlier studies also point to the absence of glycolytic activities in other Xoo species [10] and state that glycolytic activity towards carbon metabolism requires confirmation. ED (Entner-Doudoroff) pathway genes were annotated in only two of the strains studied, including the BXO43. In the Xoo IXO_1104 strain, the gene coding for Phosphogluconate dehydratase (E.C 4.2.1.12) and 2-Dehydro-3-deoxyphosphogluconate aldolase (E.C 4.1.2.14) was not annotated from the genome. These highlight that although there could be variations in the central metabolic networks in different Xoo strains, the pathways of PPP, TCA and ED are intact in BXO43. This can be further confirmed experimentally by ^13^C tracer feeding and tracking the label redistribution in the metabolites, as discussed in the following sections.

### 2.2. Xoo Cells Dependency on Nutritional Substrates

**Figure 2:**
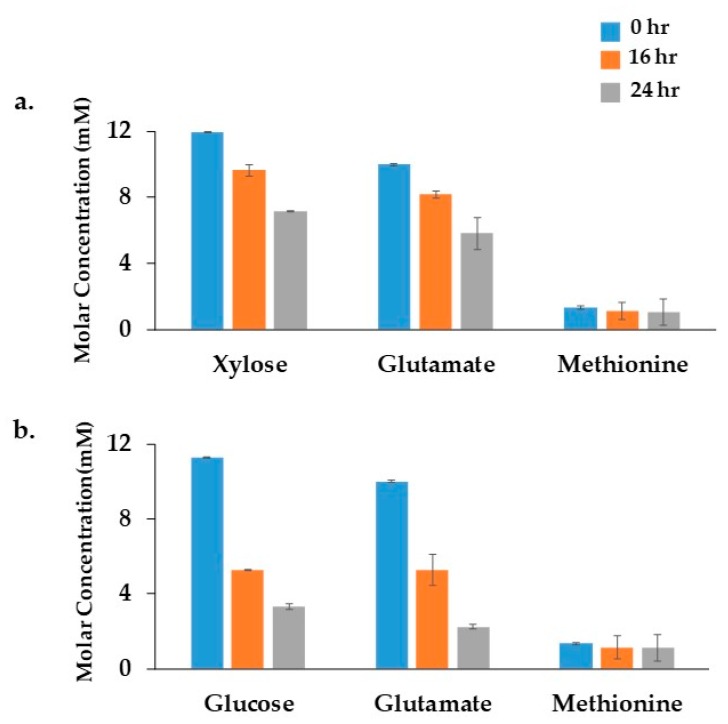
Identification of substrate consumption using ^1^H NMR: The consumption of different XOM2 media components by *Xanthomonas oryzae* pv. *oryzae* BXO43 cells was confirmed using a ^1^H NMR analysis. The *x*-axis represents the XOM2 media components with: (**a**) Xylose; and (**b**) Glucose as a carbon source. The *y*-axis indicates the molar concentration of each component in minimal media. The decrease in concentration of each component with a relevant standard error of the mean (*n* = 3) shows the consumption of Glucose, Xylose, Glutamate and Methionine by the BXO43 cells. The BXO43 cells were harvested at three different time points, namely, 0, 16 and 24 h, as depicted by the blue, orange and grey bar in each graph.

The growth of Xoo BXO43 cells under minimal media with either xylose (XOM2) or glucose was monitored for 24 h. ^1^H NMR spectra of the cell culture filtrate confirmed that Xoo cells oxidise glucose/xylose and glutamate, whereas no significant dependency of methionine was observed in both the conditions (Figure 2). Differences in growth pattern were observed in BXO43 cells fed with either glucose or xylose containing media. The initial optical density at the 0 h time point was 0.02, which reached 0.8 ± 0.05 (in glucose media) and 0.69 ± 0.04 (in xylose media) over 24 h (harvest time). In parallel, ^13^C tracers ([^13^C_6_] glucose or [^13^C_5_] xylose) were fed to the cells, which resulted in similar patterns of growth (Figure S2). The growth studies showed that BXO43 has a better glucose oxidation and glutamate uptake compared to the xylose feeding condition.

### 2.3. Accurate Assessment of Mass Isotopomer Distribution in Amino Acids

**Figure 3:**
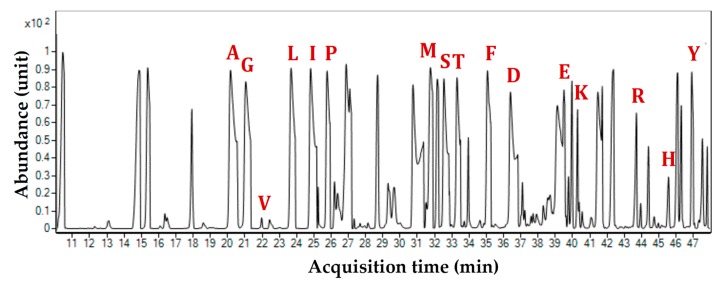
Representative total ion chromatogram (TIC) of GC-MS showing the presence of 16 amino acids from the protein hydrolysates of *Xanthomonas oryzae* pv. *oryzae* BXO43 when fed with xylose and glucose. Each peak is marked in a single letter code corresponding to the time of elution for a particular amino acid, as follows: A-Alanine; G-Glycine; V-Valine; L-Leucine; I-Isoleucine; P-Proline; M-Methionine; S-Serine; T-Threonine; P-Phenylalanine; A-Aspartate; G-Glutamate; L-Lysine; A-Arginine; H-Histidine; and T-Tyrosine. Owing to the gram-negative nature of *Xanthomonas*, additional peaks in the chromatogram are considered to be the cell wall components, obtained when performing the acid hydrolysis of the harvested Xoo samples.

The redistribution of ^13^C across the central carbon network of BXO43 cells fed with either [^13^C_6_] glucose or [^13^C_5_] xylose was successfully captured by using Gas Chromatography-Mass Spectroscopy (GC-MS) of TBDMS-derivatised proteinogenic amino acids. The Total Ion Chromatograms (TIC) resulted in 16 amino acids (Figure 3). The loss of cysteine and tryptophan due to oxidation is in accordance with the reported literature [17]. The 16 amino acids with their elution times (in minutes) are: Alanine (20.12); Glycine (21.23); Valine (22.07); Leucine (23.78); Isoleucine (25.13); Proline (25.87); Methionine (31.87); Serine (32.54); Threonine (33.25); Phenylalanine (35.05); Aspartate (36.78); Glutamate (39.98); Lysine (40.46); Arginine (43.67); Histidine (45.79) and Tyrosine (47.23). Each TBDMS-derivatised amino acid resulted in different fragments in the mass spectroscopy due to ionization and the loss of fragments [M − 0]^+^, [M − 15]^+^, [M − 85]^+^, [M − 159]^+^ and [M − R]^+^ (R denotes the side chain of an amino acid, often resulting in fragment [f302]^+^). Across the amino acid fragments, [M − 0]^+^ has shown significant deviation from the expected natural ^13^C abundance values whereas [M − 15]^+^, [M − 85]^+^, [M − 159]^+^ and [M − R]^+^ were considered further for accurate identification of incorporated ^13^C label owing to the fact that several of these fragments have mass isotopomer distributions (MIDs) much closer to the expected values. The mass ions (*m*/*z*) of each amino acid fragment and their abundances were further analysed by first correcting for the ^13^C natural abundance; the relative fractions of the corrected MIDs were then obtained. The comprehensive list of the measured and corrected MIDs for BXO43 amino acids is presented in Tables S1 and S2. These were further used to derive the corresponding average ^13^C proportions. The amino acid-derived mass isotopomer fragments were successfully validated by comparing the corrected mass isotopomer distributions (MID) with a theoretical ^13^C label in unlabelled fragments. In the case of the fragments derived from unlabelled amino acids (obtained from ^12^C tracer feeding), the fraction of ^13^C is assumed to be less than the natural abundance (i.e., <1.13%). Thus, with some relaxation for technical errors of GC-MS, amino acid fragments derived from unlabelled protein samples (BSA and Xoo cells) with an average ^13^C < 2% (except proline, which was <2.5%) were assigned as valid (Table S3). In total, 33 valid and 76 invalid mass isotopomer fragments of amino acids were obtained. It was observed that fragments such as Val302, Leu200, Leu302, Ile302, Ser302 and Asp316 were invalid, as also reported by Antoniewicz et al. [17]. None of the fragments were found to be valid for Leu, Ile, and Arg. The average ^13^C abundances (%) of amino acids pointed to the activities of the key metabolic pathways, as presented in Section 2.4. 

### 2.4. ^13^C Label Incorporation in Amino Acids Highlights the Metabolic Pathway Activities in Xoo

**Figure 4:**
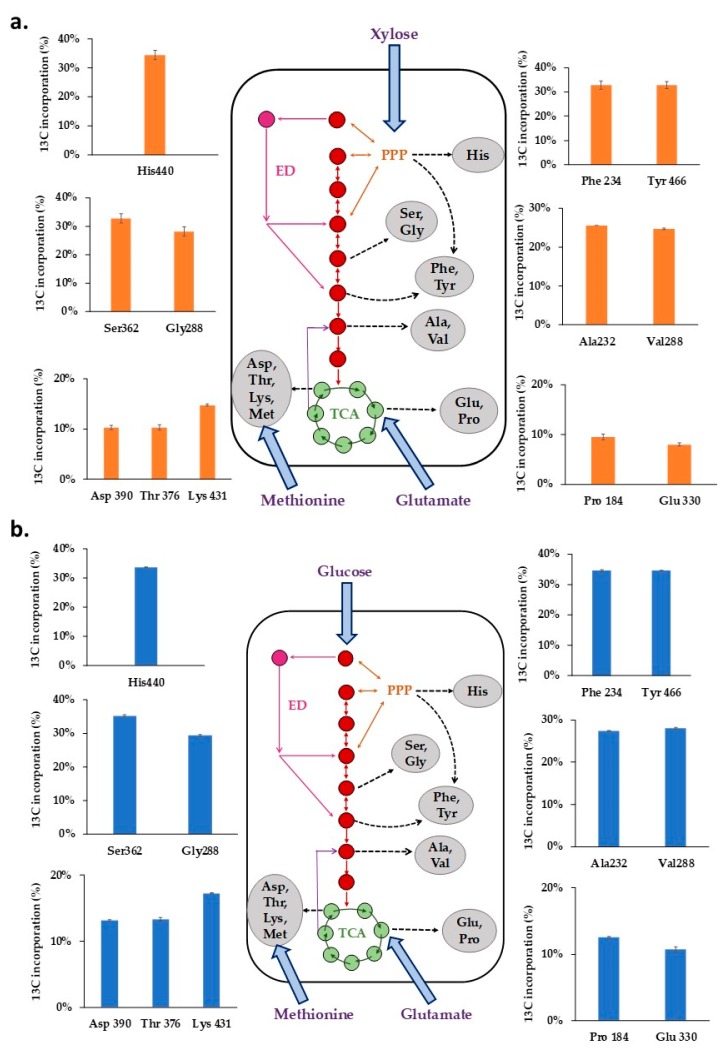
The intracellular fate of the ^13^C label in the central metabolic pathway when (**a**) 40% [^13^C_5_] xylose and (**b**) 40% [^13^C_6_] glucose was fed to *Xanthomonas oryzae* pv. *oryzae* BXO43. The average ^13^C incorporation (in %) in the representative amino acid fragments, shown as bars, highlight the activities of different metabolic pathways in Xoo. The mass isotopomer abundances of the 12 representative amino acid fragments are presented in Table S4.

The distribution of average ^13^C levels in representative amino acid fragments of Xoo BXO43 cells fed with either [^13^C_5_] xylose or [^13^C_6_] glucose (Figure 4a,b, Table S3) highlight the central metabolic pathway activities. The ^13^C label incorporation in the mass isotopomers of proteinogenic amino acids retrobiosynthetically report on the central metabolite precursors and corresponding pathways. 

When 40% [^13^C_5_] xylose was fed to Xoo BXO43, it was observed that the pentose phosphate pathway is active, as represented by the average ^13^C levels in histidine (30.63%), Phe (29.94%) and Tyr (29.76%). The average ^13^C in methionine (1.11%) confirms that it is not synthesised *denovo* and must have been assimilated from the media. The highest (30.63%) and lowest (1.11%) fraction of the average ^13^C was measured in histidine (His440) and methionine (Met320), respectively. The average ^13^C tracer incorporation in Ala (23.39%), Val (23.14%), Ser (29.32%) and Gly (25.19%) could not confirm the activity of either glycolytic/ED pathway, owing to our inability to annotate the gene for phosphoglucoisomerase in the Xoo BXO43. The activity of the TCA cycle was confirmed by the presence of ^13^C tracer in Asp (10.68%), Glu (8.34%), Lys (14.08%), Pro (9.63%) and Thr (10.63%). 

Similarly, when 40% [^13^C_6_] glucose was fed, the highest (35.43%) and lowest (1.24%) average ^13^C fractions were present in serine (Ser390) and methionine (Met320), respectively. The average ^13^C of histidine (His440, 33.9%), Phe (34.64%) and Tyr (34.68%) confirmed the activity of the pentose phosphate pathway. The average ^13^C label incorporation of Ser (35.29%), Gly (29.74%), Ala (27.45%), and Val (28.11%) could not discriminate activities of either glycolytic or ED pathway owing to our inability to annotate the gene for phosphoglucoisomerase in the Xoo BXO43. The incorporation of the ^13^C label in the Glu (8.34%), Pro (12.6%), Asp (13.14%), Thr (13.36%) and Lys (17.31%) fragments shows the active TCA (Tricarboxylic acid) cycle of the central metabolic pathway.

## 3. Discussion

Understanding the metabolic phenotypes of *Xanthomonas oryzae* (Xoo), a devastating bacterial phytopathogen, under different nutritional regimes is of interest. It has been observed that the metabolic pathways of Xoo BXO43 have not been studied in well-established plant mimic media XOM2 [12]. In this study, we qualitatively mapped and confirmed the central carbon metabolic pathways of Xoo BXO43 using [^13^C_6_] glucose or [^13^C_5_] xylose as tracers. 

An elucidation of the complete metabolic network is required for understanding the Xoo metabolism at the systems level [18]; hence, we mapped the annotated genes and evaluated their pathways. First, we comparatively analysed the central metabolic networks of three selected Xoo strains using the genome annotations and KEGG mapper tool (Figure 1). The presence of several pathways like PPP, TCA, ED and amino acid biosynthesis was established in Xoo BXO43 and was in agreement with other bacterial species [19,20,21,22,23,24]. One variation noted was the absence of any annotated gene coding for the key glycolytic enzyme, phosphoglucoisomerase, in Xoo BXO43, which might reflect variations of metabolic features among different Xoo strains. Phosphoglucoisomerase (PGI), or glucose-6-phosphate isomerase (GPI), is the potential enzyme of the glycolytic route responsible for the interconversion of glucose-6-phosphate and fructose-6-phosphate. It is possible that bacterial species lack few glycolytic enzymes and would prefer pentose phosphate (PPP) and ED pathways for fulfilling the metabolic demands of the cells [25,26,27]. There are discrepancies in literature about the phosphoglucoisomerase in Xoo. Tsuge et al. [16] reported that *Xanthomonas oryzae* pathogenicity is directly related to the induction of PGI gene expression under xylose containing minimal media. However, from the available whole genome sequences of different Xoo strains [3], when annotated, the PGI gene was not found in Xoo BXO43 while it was present in the other two stains studied (Figure 1). BXO43 is known to exhibit pathogenicity in rice, which brings into question the potential contribution of PGI to infection and metabolism. Although the KEGG mapper allowed us to overlay the annotated genes of the central metabolic pathways of Xoo, it did not necessarily confirm the pathway activities. Therefore, we undertook ^13^C tracer feeding to map the pathway activities.

The oxidation of [^13^C_6_] glucose and [^13^C_6_] xylose in Xoo BXO43 resulted in the incorporation of ^13^C in amino acids which retro-biosynthetically reports on the labelling of central metabolite precursors and the activities of corresponding pathways [28]. The ^13^C label redistribution of the metabolites in the case of xylose feeding represents the in vivo host conditions, i.e., the plant mimicked minimal media (XOM2) as determined by Tsuge et al. [12]. It could be of immense relevance to infer metabolic responses under such pathogenic conditions. Similarly, the ^13^C glucose feeding could be representative of the free-living conditions of Xoo. We conducted a GC-MS-based mass isotopomer analysis of the resulting proteinogenic amino acids to obtain insights into the activities of the central metabolic pathways. First, we comprehensively established reliable mass isotopomer fragments that were successfully used for tracking the label incorporation and in quantifying the average ^13^C [29,30] in amino acids that report on different pathway activities (Section 2.4). The major central metabolic pathways whose activities were confirmed based on ^13^C labelling included PPP (based on histidine), and the TCA cycle (based on labelling in Asp, Glu, Pro and Lys). Although Alanine, glycine and serine are labelled, this could not confirm the activities of glycolytic, ED or other pathways owing to the multiple routes of biosynthesis. The average ^13^C of the amino acids derived from the TCA cycle (Asp, Glu, Pro and Lys) and lower glycolysis (Ala, Ser, Gly) were found to be less than histidine, which could potentially be explained by the uptake of unlabelled glutamate contributing to the TCA cycle and anaplerotic reactions. The almost negligible ^13^C incorporation in methionine brings into question its potential role in BXO43 carbon metabolism. It may play a significant role in the induction of hrp (hypersensitive reaction and pathogenicity) gene expression, which was earlier reported in XOM2 minimal media [12]. 

The similar patterns of ^13^C labelling of amino acids (His, Ala, Ser, Val and Gly) between glucose and xylose feeding experiments (Figure 4) plausibly suggests that the C1 of glucose is lost as CO_2_ via the oxidative PPP (OPPP), predominantly. The rest of the five carbons (C2–C6) are oxidised via the PPP-generating histidine and other central precursors (GAP and F6P), which further enter the lower glycolytic pathways and the TCA cycle, similar to the case of xylose feeding. Based on the current analysis, it can be predicted that PPP is the main metabolic route in Xoo BXO43. The glycolytic pathways, owing to the possible absence (unannotated) of the PGI gene in the *Xanthomonas oryzae* BXO43 strain, seem inactive. However, this requires further confirmation using the positional labelling experiments. Moreover, the differences in the TCA cycle labelling (Asp, Glu and Pro) in glucose and xylose feeding can be attributed to the differences in metabolism (anaplerotic pathways) and the varying levels of glutamate uptake under different substrates. 

The extent of the average ^13^C labelling in all the amino acids was less than the maximum expected values of 40% (the average ^13^C level of the substrates fed was 40%). This highlights the contribution of the pre-existing biomass and/or other potential unlabelled carbon sources in culture media (glutamate, methionine) or carbon dioxide incorporation. The contributions of these unlabelled carbon need to be accounted for during future flux analysis studies. This study provided the first qualitative insights into central carbon metabolic pathway activities in Xoo. Furthermore, it mapped the pathways and provided metabolic insights into the features of *Xanthomonas oryzae* (Xoo) BXO43 in plant mimicking XOM2 media. In addition, it points to the need to use optimal tracer substrates ([1-^13^C]glucose/ [1-^13^C]xylose) for deriving Xoo flux maps that would eventually define the metabolic phenotypes of this important phytopathogen.

## 4. Materials and Methods

### 4.1. KEGG Mapper-Based Metabolic Pathway Reconstruction

KEGG (Kyoto Encyclopedia of Genes and Genomes) Mapper Version 3.0, a pathway mapping tool available online was employed for the metabolic pathway generation of three *Xanthomonas oryzae* strains (BXO43, IXO_1088 and IXO_1104). The publicly available genome annotation data files of the selected Xoo strains from Midha et al. [3] were uploaded onto the Blast koala software [31] to extract the corresponding KEGG Orthology (KO) numbers. The gene lists with all the KO annotations were then entered into the KEGG mapper tool to map the pathways. The gene annotations corresponding to the central metabolic pathways were obtained, which also resulted in the comparison of the three Xoo strains.

### 4.2. Xanthomonas oryzae Strain Maintenance

The wild-type *Xanthomonas oryzae* pv. *oryzae* BXO43 (Xoo) strain was obtained from the Centre for Cellular and Molecular Biology (CCMB), Hyderabad, and used for all the experimental work. Peptone sucrose (PS) containing rich media with or without Agar and rifampicin (5 mg/mL) was used for the routine maintenance and cultivation of BXO43 at 28 °C at a pH of 7.2 [8]. On PS (5 g peptone and 5 g sucrose per litre) Agar, the Xoo colonies appeared between 3–5 days. For liquid cultures, the cells were incubated at 28 °C with shaking at 180 rpm for 6–7 days. The glycerol stocks of Xoo were prepared and maintained at −80 °C. 

### 4.3. Growth Experiments of Xoo

For all the feeding experiments, the Xoo BXO43 cells were first cultivated at 28 °C with shaking at 180 rpm in a nutrient-rich PS broth until the OD_600_ reached 0.5; they were then washed twice using XOM2 media without a carbon source and immediately transferred into the XOM2 media with either xylose or glucose. The growth pattern of the BXO43 cells in defined minimal media (XOM2) containing ^13^C tracers (40% [^13^C_6_] glucose or 40% [^13^C_5_] xylose) was monitored for 24 h, as depicted in the growth curve (Figure S2). XOM2, a known plant mimic media [12], consisted of 670 µM d, l-methionine, 10 mM sodium l-(+)-glutamate, 14.7 mM KH_2_PO_4_, 40 µM MnSO_4_, 240 µM Fe(III) EDTA, 5 mM MgCl_2_ and either 0.18% xylose or glucose, pH 7.2 [8,12]. The ^13^C enrichment of the glucose or xylose was 40% (i.e., 0.18% of (40% [^13^C_6_] glucose + 60% unlabelled glucose) or 0.18% of (40% [^13^C_5_] xylose + 60% unlabelled xylose)). At the mid-exponential growth phase (OD_600_ of 0.4–0.8), the culture filtrate from the incubated Xoo cells was harvested for further ^1^H NMR analysis to determine the consumption of nutrient tracers by *Xoo* cells. The cell pellets were harvested for subsequent acid hydrolysis and the GC-MS analysis of the proteinogenic amino acids for ^13^C incorporation. All the media components were obtained from Himedia and Cambridge isotope laboratories. The carbon sources and the media components were sterilised using syringe filters.

### 4.4. Nuclear Magnetic Resonance (^1^H NMR) of Culture Filtrate

A total of 50 µL harvested bacterial culture filtrate was mixed with 900 µL of D_2_O and vortexed for 10 min (*n* = 3). After bringing it to room temperature, 100 µL of 0.1% DSS (4,4-dimethyl-4-silapentane-1-sulfonic acid) prepared in D_2_0 was added to each sample as an internal standard. Subsequently, the tubes were centrifuged at 13,000 rpm for 10 min at 4 °C. A total of 600 µL aliquots of culture filtrate were then transferred to 5 mm NMR tubes for data acquisition. ^1^H NMR spectra were recorded at 19 °C on a 500 MHz JEOL ECX-500 spectrometer in the Advanced Materials Research Centre facility of IIT Mandi. Each ^1^H NMR data consisted of 32 scans requiring an acquisition time of 10 min with the following parameters: pulse width (PW) = 11.6 [µs] and relaxation delay (RD) = 5.0 s. A presaturation sequence was used to suppress the residual H_2_O signal with low power selective irradiation at the H_2_O frequency during the recycle delay. The resulting spectra were manually phased and the baseline corrected and calibrated to DSS (4,4-dimethyl-4-silapentane-1-sulfonic acid) at 0.0 ppm, using ECX NMR (version 3.5, JEOL, Tokyo, Japan). 

The recorded ^1^H NMR spectra were automatically reduced to ASCII files. The spectral intensities were scaled to the internal standard and the integration calculated for the glucose (4.62–4.65 ppm), xylose (3.9–3.94 ppm), glutamate (3.6–3.72) and methionine (1.7–1.8) peaks using Delta software (Version 5.5). The regions from 0–0.2, 0.61–0.64, 1.72–1.79 and 2.8–2.9 ppm were excluded from the analysis as these correspond to the signal from the internal standard (DSS). The calculated integration values were then correlated to the concentration of carbon tracer (glucose and xylose) and XOM2 media components (glutamate and methionine) at 0, 16 and 24 h time points to measure their consumption by Xoo cells. 

### 4.5. Cell Hydrolysis

Harvested bacterial cell pellets (4 mg) were acid hydrolysed by suspending them in 500 µL 6M HCl and then transferred to screw-capped cryogenic tubes. The tubes were partially sealed and placed in a heat block inside the fume hood cabinet for 20 h at 100 °C [17]. The hydrolysate was subjected to centrifugation at 13,000× *g* for 10 min to separate the water-soluble amino acids from insoluble pellets. A total final volume of about 140 µL was obtained by adding water for homogenous levels in all tubes. A total of 100 µL of supernatant from each replicate was collected into a fresh tube and vacuum-dried at 40 °C for 6 h in a speed-vacuum system to ensure the complete removal of water (Thermo-scientific, Waltham, MA, USA). The dried hydrolysates were subjected to derivatisation for gas chromatography and mass spectrometry.

### 4.6. GC-MS Analysis of TBDMS-Derivatised Amino Acids

#### 4.6.1. Derivatisation of Proteinogenic Amino Acids

The dried protein hydrolysates were subjected to TBDMS derivatisation [32,33,34] and subsequent GC-MS analysis [35,36]. To each dried extract, 30 μL of pyridine was added and the tubes were incubated at 37 °C for 30 min on a thermomixer set at 900 rpm. Then, to each tube 50 μL of MTBSTFA (*N*-Methyl-*N*-(t-butyldimethylsilyl trifluoroacetamide)) + 1% t-BDMCS(t-butyl dimethylchlorosilane) was added followed by incubation at 60 °C for 30 min in thermomixer at 900 rpm [15]. Subsequently, the samples were centrifuged at 13,000 × *g* for 10 min and the supernatant was transferred to 200 μL glass vial inserts and subjected to GC-MS. The derivatising reagents were obtained from Sigma Aldrich. 

#### 4.6.2. Processing of GC-MS Spectra

##### Data Acquisition

The derivatised protein hydrolysates of *Xanthomonas oryzae* were placed in an automatic liquid sampler (7683B ALS, Agilent Technologies, Santa Clara, CA, USA) and 2 μL was injected into the DBX-5 column using the splitless mode for GC−MS (7890A GC, 5975 MSD from Agilent Technologies, USA) acquisition at the facility of IIT Roorkee. Helium was used as a carrier gas at a constant flow rate of 1.3 mL/min. The temperature of the front injector was operated isothermally at 230 °C. The temperature gradient of 5 min at 120 °C isothermal was used, followed by a 4 °C min^−1^ oven temperature gradient up to a final 270 °C, and then held for 3 min at 270 °C, followed by a 20 °C min^−1^ up to a final 320 °C temperature for 1 min. Ions were generated using a 70 eV electron beam in electron ionization mode. The spectra were recorded with a scanning range of 50–600 mz^−1^ for a total run time of 50 min. Chemstation software (Agilent Technologies, USA) was used to control the data acquisition parameters (both GC separation and mass spectrometry) during all the sample runs. 

##### Mass Spectral Data Pre-Treatment

The baseline correction was applied to all the raw GC-MS data files to estimate the accurate mass isotopomer distribution (MID) for all amino acids. The baseline correction of the recorded GC-MS spectra was carried out using Metalign software [37]. The peaks of the baselined spectra were identified using chemstation software based on the *m*/*z* of different fragments, their elution times and hits against the NIST (National Institute of Standards and Technology, Maryland) Library and the amino acid standards. The intensity of the mass ions of each amino acid fragment [38] was obtained by using Agilent chemstation software. The fragment details are presented in Section 2.3. The MIDs were then tabulated for further natural isotope correction. 

#### 4.6.3. Isocorr-Based Natural Isotope (^13^C) Abundance Correction and Average ^13^C Calculation

The natural abundance of ^13^C isotope contributes to the measured mass isotopomers of all amino acid fragments [39]. The correction of stable isotope abundance is a prerequisite for accurate quantification of mass isotopomer abundances from the supplied ^13^C carbon tracer [40]. The GC-MS-derived amino acid fragments as well as the ^13^C incorporation in derivatisation reagents was corrected using a well-established algorithm of Isocorr software [41]. The derivatisation and metabolite formula required for Isocorr was calculated manually for all the amino acid fragments. The MID values corrected for stable isotope abundance were finally subjected to calculate the average ^13^C abundance in each fragment. The average ^13^C proportion for each amino acid fragment was calculated as per the following equation [14]:

$$\mathrm{Average}{\;}^{13}C\;=\;(\sum_{i=0}^{N}n_{m+i}\times A)/N$$

where *n_m + i_* is number of ^13^C containing carbons in the mass isotopomer (*m* + *i*). e.g., *n* = 0 for *m* + 0, *n* = 1 for *m* + 1 and so on until *i* = total number of carbons (N) in the fragment; and A is the corresponding fractional abundance of the mass isotopomer.

## Supplementary Materials

The following are available online at http://www.mdpi.com/2218-1989/8/4/66/s1: Figure S1: Experimental workflow showing the cultivation of *Xanthomonas oryzae* pv. *oryzae* BXO43 cells followed by isotopic tracer feeding and GC-MS data analysis towards average ^13^C label incorporation in proteinogenic amino acids; Figure S2: The growth curve of *Xanthomonas oryzae* pv. *oryzae* BXO43 cells in XOM2 minimal media containing 0.18% (*w*/*v*) glucose or xylose as carbon source. The growth is observed for 24 h (*n* = 3) for both the conditions; Table S1: Measured and corrected Mass isotopomer distributions (MID) of amino acid fragments of *Xanthomonas oryzae* pv. *oryzae* BXO43 grown in XOM2 minimal media containing 40% [^13^C_6_] glucose; Table S2: Measured and corrected Mass isotopomer distributions (MID) of amino acid fragments of *Xanthomonas oryzae* pv. *oryzae* BXO43 grown in XOM2 minimal media containing 40% [^13^C_5_] xylose; Table S3: Average ^13^C abundance (in %) in amino acid fragments of *Xanthomonas oryzae* pv. *oryzae* BXO43 subjected to minimal media containing 40% [^13^C_6_] glucose or 40% [^13^C_5_] xylose; Table S4: The Mass Isotopomer Distributions (MID) of 12 representative valid amino acid fragments in central metabolic pathway of *Xanthomonas oryzae* pv. *oryzae* BXO43.
