# Supplementary material for: Intracellular Fate of Universally Labelled 13C Isotopic Tracers of Glucose and Xylose in Central Metabolic Pathways of Xanthomonas oryzae
Source: Metabolites. 2018 Oct 15;8(4):66. doi: 10.3390/metabo8040066 (PMC6316632; doi:10.3390/metabo8040066)
Supplement: Supplementary file 1 [file metabolites-08-00066-s001.zip › Final Edited Supplementary/Supplementary Table S4.docx]

**Supplementary Table S4:** The Mass Isotopomer Distributions (MID) of 12 representative valid amino acid fragments in central metabolic pathway of *Xanthomonas oryzae* pv. *oryzae* BXO43. All the MIDs are presented along with the standard deviation from three technical replicate in each condition. The average 13C incorporation of these fragments are depicted in Figure 4.

| **Metabolite fragment** | **Fragment ion** | **Carbon numbers** | **Mass Isotopomers** | **Unlabelled**  **Glucose** | **40% [^13^C_6_]**  **Glucose** | **Unlabelled**  **Xylose** | **40% [^13^C_5_]**  **Xylose** |
| --- | --- | --- | --- | --- | --- | --- | --- |
| **Ala232** | [M-85]^+^ | 2,3 | m0 | 0.963 ± 0.001 | 0.663 ± 0.001 | 0.962 ± 0.002 | 0.711 ± 0.054 |
|  |  |  | m1 | 0.035 ± 0.001 | 0.125 ± 0.001 | 0.036 ± 0.002 | 0.110 ± 0.019 |
|  |  |  | m2 | 0.001 ± 0.000 | 0.212 ± 0.001 | 0.001 ± 0.000 | 0.179 ± 0.036 |
| **Val288** | [M-57]^+^ | 1,2,3,4,5 | m0 | 0.980 ± 0.001 | 0.440 ± 0.004 | 0.980 ± 0.001 | 0.510 ± 0.075 |
|  |  |  | m1 | 0.018 ± 0.001 | 0.119 ± 0.001 | 0.019 ± 0.001 | 0.121 ± 0.018 |
|  |  |  | m2 | 0.001 ± 0.000 | 0.186 ± 0.001 | 0.001 ± 0.000 | 0.175 ± 0.031 |
| **Ser362** | [M-85]^+^ | 2,3 | m0 | 0.964 ± 0.001 | 0.533 ± 0.005 | 0.964 ± 0.002 | 0.620 ± 0.085 |
|  |  |  | m1 | 0.035 ± 0.001 | 0.228 ± 0.003 | 0.035 ± 0.002 | 0.174 ± 0.035 |
|  |  |  | m2 | 0.000 ± 0.000 | 0.239 ± 0.002 | 0.000 ± 0.000 | 0.206 ± 0.054 |
| **Asp390** | [M-85]^+^ | 2,3,4 | m0 | 0.959 ± 0.001 | 0.728 ± 0.002 | 0.959 ± 0.001 | 0.784 ± 0.009 |
|  |  |  | m1 | 0.040 ± 0.001 | 0.168 ± 0.001 | 0.041 ± 0.001 | 0.128 ± 0.006 |
|  |  |  | m2 | 0.000 ± 0.000 | 0.086 ± 0.001 | 0.000 ± 0.000 | 0.071 ± 0.005 |
|  |  |  | m3 | 0.000 ± 0.000 | 0.018 ± 0.001 | 0.000 ± 0.016 | 0.000 ± 0.006 |
| **Thr376** | [M-85]^+^ | 2,3,4 | m0 | 0.958 ± 0.006 | 0.724 ± 0.005 | 0.957 ± 0.003 | 0.783 ± 0.012 |
|  |  |  | m1 | 0.042 ± 0.006 | 0.169 ± 0.004 | 0.042 ± 0.002 | 0.130 ± 0.014 |
|  |  |  | m2 | 0.000 ± 0.001 | 0.088 ± 0.002 | 0.000 ± 0.001 | 0.072 ± 0.005 |
|  |  |  | m3 | 0.000 ± 0.000 | 0.018 ± 0.000 | 0.000 ± 0.000 | 0.015 ± 0.007 |
| **Lys431** | [M-R]^+^ | 1,2,3,4,5,6 | m0 | 0.926 ± 0.001 | 0.485 ± 0.003 | 0.927 ± 0.002 | 0.567 ± 0.046 |
|  |  |  | m1 | 0.074 ± 0.001 | 0.180 ± 0.000 | 0.072 ± 0.001 | 0.156 ± 0.013 |
|  |  |  | m2 | 0.001 ± 0.001 | 0.180 ± 0.001 | 0.001 ± 0.001 | 0.160 ± 0.025 |
|  |  |  | m3 | 0.000 ± 0.000 | 0.124 ± 0.002 | 0.000 ± 0.000 | 0.099 ± 0.011 |
|  |  |  | m4 | 0.000 ± 0.000 | 0.032 ± 0.001 | 0.000 ± 0.000 | 0.018 ± 0.003 |
|  |  |  | m5 | 0.000 ± 0.000 | 0.000 ± 0.000 | 0.000 ± 0.000 | 0.000 ± 0.000 |
|  |  |  | m6 | 0.000 ± 0.000 | 0.000 ± 0.000 | 0.000 ± 0.000 | 0.000 ± 0.000 |
| **His440** | [M-57]^+^ | 1,2,3,4,5,6 | m0 | 0.921 ± 0.008 | 0.278 ± 0.010 | 0.921 ± 0.004 | 0.372 ± 0.141 |
|  |  |  | m1 | 0.068 ± 0.004 | 0.186 ± 0.003 | 0.067 ± 0.003 | 0.188 ± 0.020 |
|  |  |  | m2 | 0.005 ± 0.002 | 0.144 ± 0.002 | 0.005 ± 0.001 | 0.110 ± 0.031 |
|  |  |  | m3 | 0.004 ± 0.002 | 0.171 ± 0.003 | 0.004 ± 0.001 | 0.106 ± 0.034 |
|  |  |  | m4 | 0.001 ± 0.001 | 0.097 ± 0.001 | 0.002 ± 0.000 | 0.059 ± 0.018 |
|  |  |  | m5 | 0.000 ± 0.000 | 0.087 ± 0.002 | 0.001 ± 0.001 | 0.112 ± 0.031 |
|  |  |  | m6 | 0.000 ± 0.000 | 0.037 ± 0.000 | 0.000 ± 0.000 | 0.053 ± 0.012 |
| **Glu330** | [M-85]^+^ | 2,3,4,5 | m0 | 0.947 ± 0.001 | 0.736 ± 0.011 | 0.947 ± 0.001 | 0.796 ± 0.010 |
|  |  |  | m1 | 0.050 ± 0.000 | 0.130 ± 0.005 | 0.051 ± 0.000 | 0.099 ± 0.004 |
|  |  |  | m2 | 0.002 ± 0.000 | 0.102 ± 0.005 | 0.002 ± 0.000 | 0.083 ± 0.004 |
|  |  |  | m3 | 0.000 ± 0.000 | 0.027 ± 0.001 | 0.000 ± 0.000 | 0.017 ± 0.004 |
|  |  |  | m4 | 0.000 ± 0.000 | 0.005 ± 0.000 | 0.000 ± 0.000 | 0.004 ± 0.004 |
| **Pro184** | [M-159]^+^ | 2,3,4,5 | m0 | 0.927 ± 0.006 | 0.700 ± 0.003 | 0.929 ± 0.007 | 0.773 ± 0.008 |
|  |  |  | m1 | 0.058 ± 0.002 | 0.143 ± 0.001 | 0.058 ± 0.003 | 0.108 ± 0.005 |
|  |  |  | m2 | 0.008 ± 0.001 | 0.119 ± 0.001 | 0.007 ± 0.001 | 0.088 ± 0.002 |
|  |  |  | m3 | 0.002 ± 0.001 | 0.031 ± 0.000 | 0.002 ± 0.001 | 0.020 ± 0.002 |
|  |  |  | m4 | 0.005 ± 0.002 | 0.007 ± 0.000 | 0.004 ± 0.002 | 0.010 ± 0.004 |
| **Phe234** | [M-159]^+^ | 2,3,4,5,6,7,8,9 | m0 | 0.899 ± 0.003 | 0.216 ± 0.007 | 0.901 ± 0.001 | 0.267 ± 0.131 |
|  |  |  | m1 | 0.090 ± 0.001 | 0.086 ± 0.001 | 0.090 ± 0.001 | 0.131 ± 0.017 |
|  |  |  | m2 | 0.007± 0.000 | 0.203 ± 0.003 | 0.007 ± 0.001 | 0.175 ± 0.034 |
|  |  |  | m3 | 0.001 ± 0.001 | 0.125 ± 0.002 | 0.001 ± 0.001 | 0.126 ± 0.035 |
|  |  |  | m4 | 0.000 ± 0.000 | 0.148 ± 0.001 | 0.000 ± 0.000 | 0.124 ± 0.027 |
|  |  |  | m5 | 0.000 ± 0.000 | 0.083 ± 0.001 | 0.000 ± 0.000 | 0.074 ± 0.019 |
|  |  |  | m6 | 0.000 ± 0.001 | 0.093 ± 0.001 | 0.000 ± 0.000 | 0.069 ± 0.012 |
|  |  |  | m7 | 0.000 ± 0.000 | 0.021 ± 0.000 | 0.000 ± 0.000 | 0.018 ± 0.003 |
|  |  |  | m8 | 0.002 ± 0.002 | 0.024 ± 0.000 | 0.001 ± 0.001 | 0.017 ± 0.001 |
| **Tyr466** | [M-57]+ | 1,2,3,4,5,6,7,8,9 | m0 | 0.893 ± 0.005 | 0.211 ± 0.004 | 0.896 ± 0.002 | 0.252 ± 0.138 |
|  |  |  | m1 | 0.098 ± 0.003 | 0.082 ± 0.001 | 0.098 ± 0.001 | 0.128 ± 0.012 |
|  |  |  | m2 | 0.005 ± 0.001 | 0.119 ± 0.001 | 0.004 ± 0.002 | 0.125 ± 0.024 |
|  |  |  | m3 | 0.001 ± 0.001 | 0.178 ± 0.003 | 0.001 ± 0.001 | 0.152 ± 0.040 |
|  |  |  | m4 | 0.001 ± 0.001 | 0.127 ± 0.000 | 0.001 ± 0.000 | 0.119 ± 0.031 |
|  |  |  | m5 | 0.001 ± 0.000 | 0.097 ± 0.000 | 0.000 ± 0.000 | 0.082 ± 0.018 |
|  |  |  | m6 | 0.000 ± 0.000 | 0.092 ± 0.001 | 0.000 ± 0.000 | 0.072 ± 0.017 |
|  |  |  | m7 | 0.000 ± 0.000 | 0.050 ± 0.001 | 0.000 ± 0.000 | 0.038 ± 0.007 |
|  |  |  | m8 | 0.001 ± 0.001 | 0.019 ± 0.000 | 0.000 ± 0.000 | 0.017 ± 0.003 |
|  |  |  | m9 | 0.001 ± 0.001 | 0.024 ± 0.001 | 0.000 ± 0.000 | 0.013 ± 0.002 |
| **Gly288** | [M-15]^+^ | 1,2 | m0 | 0.982 ± 0.012 | 0.668 ± 0.007 | 0.996 ± 0.004 | 0.683 ± 0.067 |
|  |  |  | m1 | 0.018 ± 0.012 | 0.065 ± 0.001 | 0.004 ± 0.004 | 0.130 ± 0.017 |
|  |  |  | m2 | 0.000 ± 0.000 | 0.266 ± 0.008 | 0.000 ± 0.000 | 0.187 ± 0.052 |
