# Supplementary material for: Intracellular Fate of Universally Labelled 13C Isotopic Tracers of Glucose and Xylose in Central Metabolic Pathways of Xanthomonas oryzae
Source: Metabolites. 2018 Oct 15;8(4):66. doi: 10.3390/metabo8040066 (PMC6316632; doi:10.3390/metabo8040066)
Supplement: Supplementary file 1 [file metabolites-08-00066-s001.zip › Final Edited Supplementary/Supplementary Figures revised.pptx]

## Slide 1
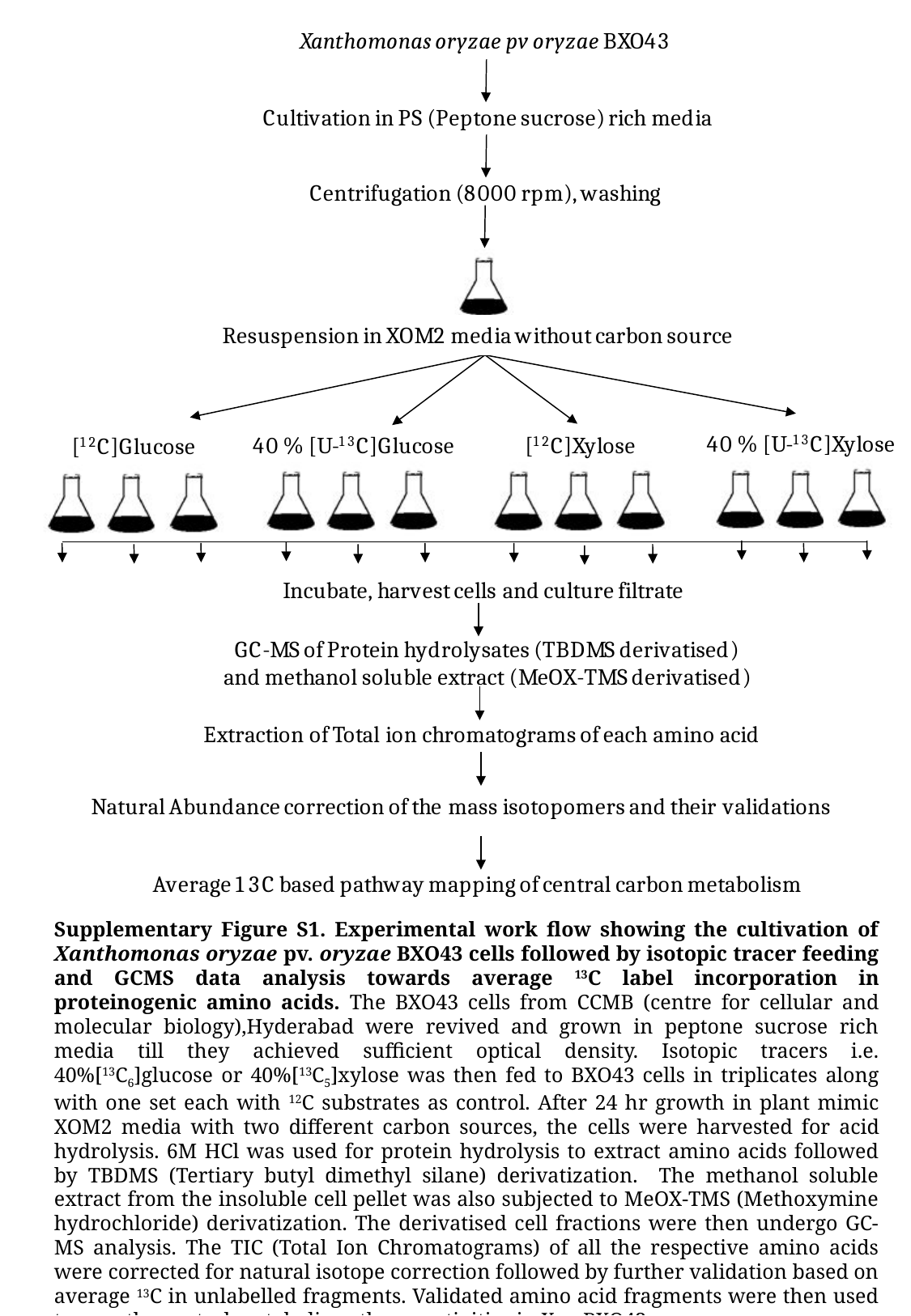

Supplementary Figure S1. Experimental work flow showing the cultivation of Xanthomonas oryzae pv. oryzae BXO43 cells followed by isotopic tracer feeding and GCMS data analysis towards average 13C label incorporation in proteinogenic amino acids. The BXO43 cells from CCMB (centre for cellular and molecular biology),Hyderabad were revived and grown in peptone sucrose rich media till they achieved sufficient optical density. Isotopic tracers i.e. 40%[13C6]glucose or 40%[13C5]xylose was then fed to BXO43 cells in triplicates along with one set each with 12C substrates as control. After 24 hr growth in plant mimic XOM2 media with two different carbon sources, the cells were harvested for acid hydrolysis. 6M HCl was used for protein hydrolysis to extract amino acids followed by TBDMS (Tertiary butyl dimethyl silane) derivatization. The methanol soluble extract from the insoluble cell pellet was also subjected to MeOX-TMS (Methoxymine hydrochloride) derivatization. The derivatised cell fractions were then undergo GC-MS analysis. The TIC (Total Ion Chromatograms) of all the respective amino acids were corrected for natural isotope correction followed by further validation based on average 13C in unlabelled fragments. Validated amino acid fragments were then used to map the central metabolic pathway activities in Xoo BXO43.

## Slide 2
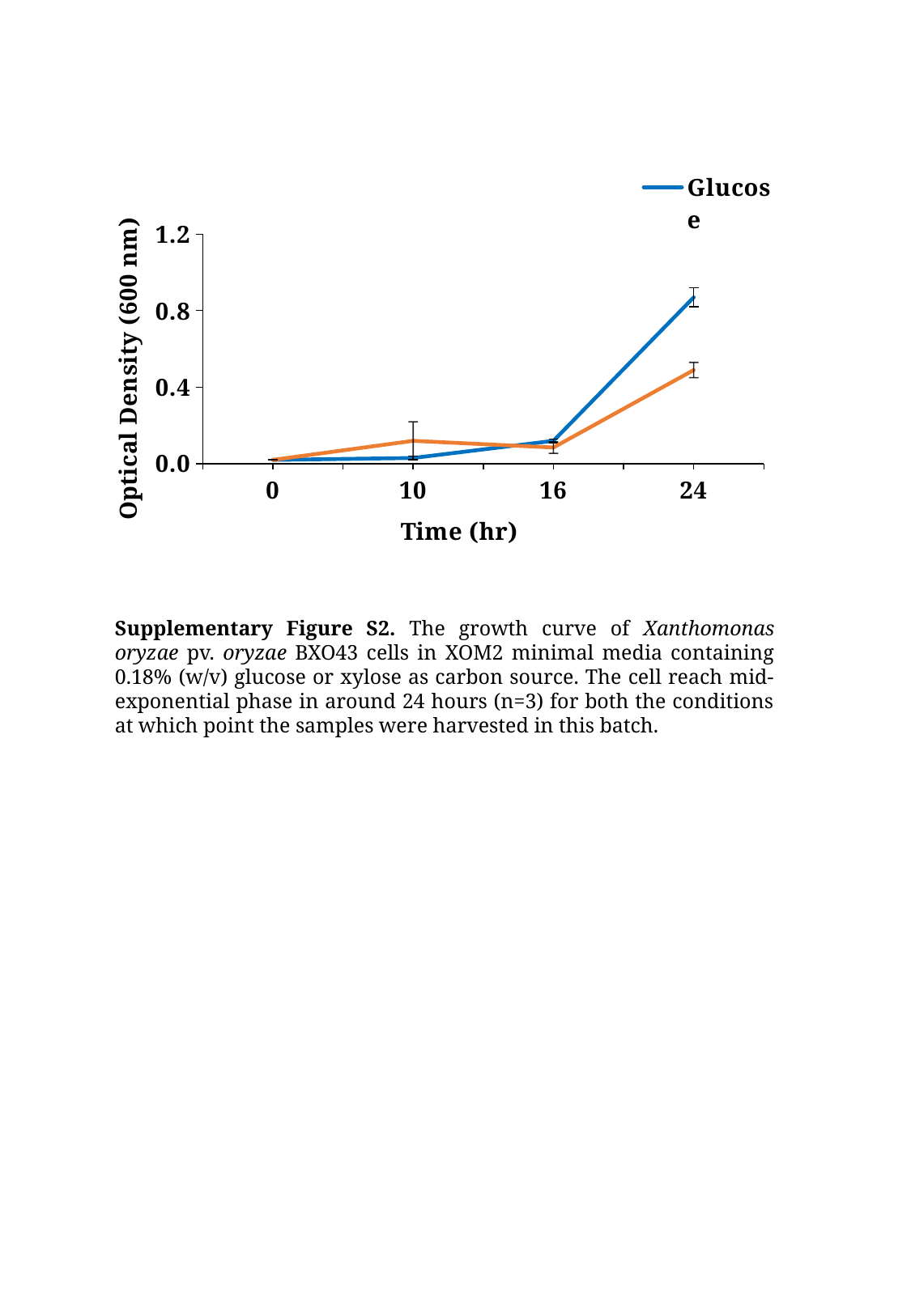

### Chart
| Category | Glucose | Xylose |
|---|---|---|
| 0 | 0.02 | 0.02 |
| 10 | 0.03 | 0.12 |
| 16 | 0.12 | 0.085 |
| 24 | 0.87 | 0.49 |Supplementary Figure S2. The growth curve of Xanthomonas oryzae pv. oryzae BXO43 cells in XOM2 minimal media containing 0.18% (w/v) glucose or xylose as carbon source. The cell reach mid-exponential phase in around 24 hours (n=3) for both the conditions at which point the samples were harvested in this batch.
